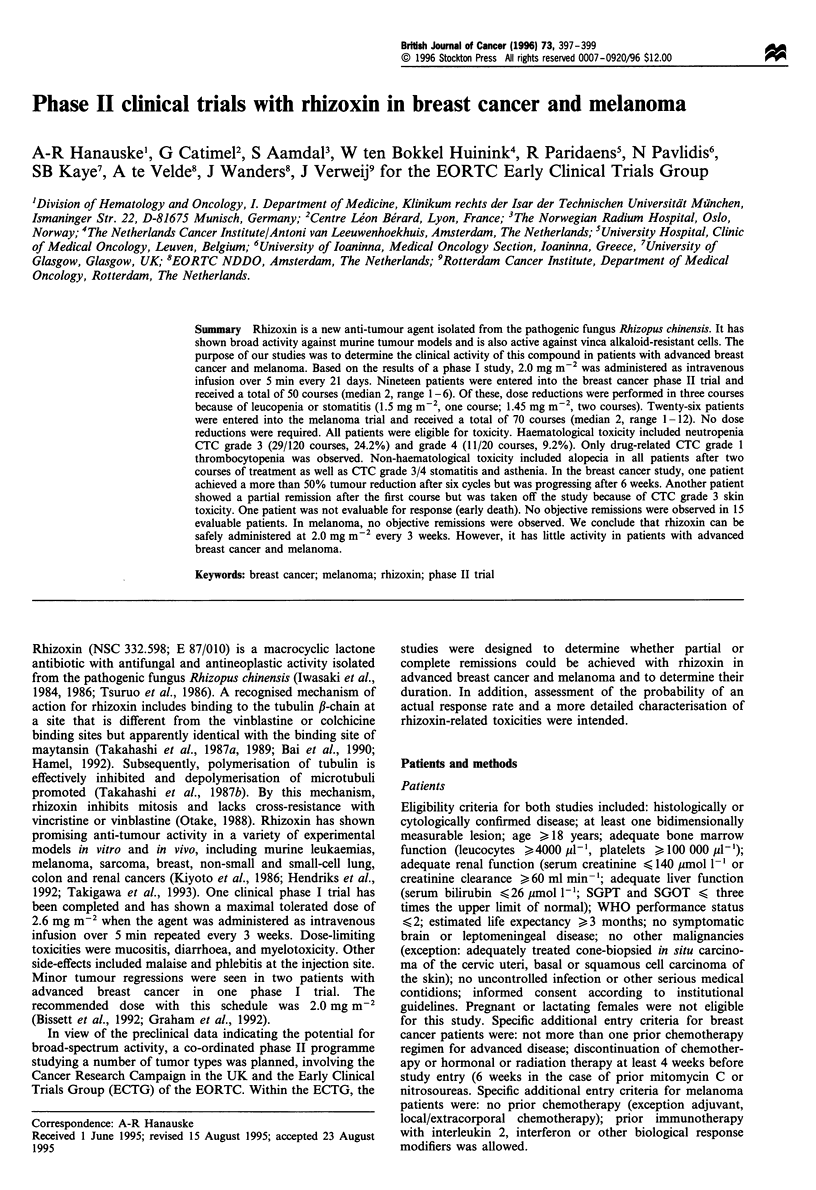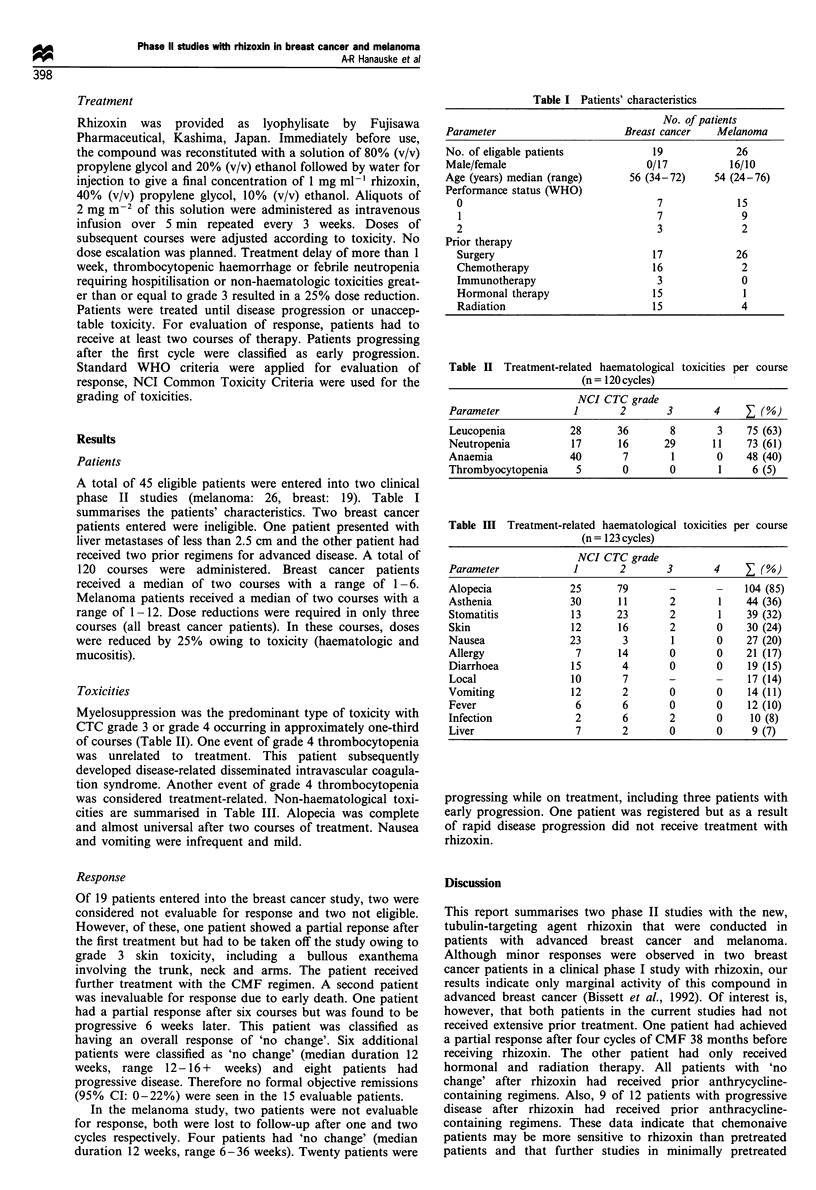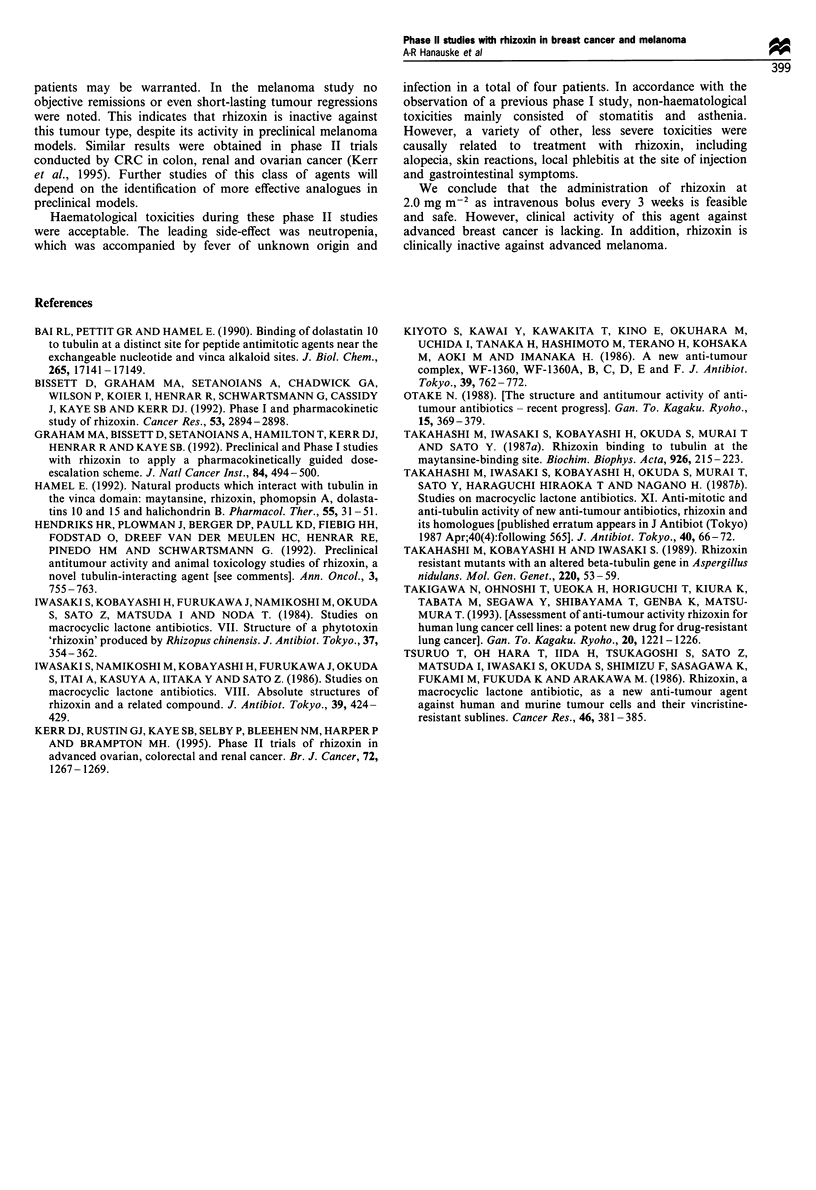# Phase II clinical trials with rhizoxin in breast cancer and melanoma. The EORTC Early Clinical Trials Group.

**DOI:** 10.1038/bjc.1996.68

**Published:** 1996-02

**Authors:** A. R. Hanauske, G. Catimel, S. Aamdal, W. ten Bokkel Huinink, R. Paridaens, N. Pavlidis, S. B. Kaye, A. te Velde, J. Wanders, J. Verweij

**Affiliations:** I. Department of Medicine, Klinikum rechts, Isar der Technischen Universität München, Germany.

## Abstract

Rhizoxin is a new anti-tumour agent isolated from the pathogenic fungus Rhizopus chinensis. It has shown broad activity against murine tumour models and is also active against vinca alkaloid-resistant cells. The purpose of our studies was to determine the clinical activity of this compound in patients with advanced breast cancer and melanoma. Based on the results of a phase I study, 2.0 mg m-2 was administered as intravenous infusion over 5 min every 21 days. Nineteen patients were entered into the breast cancer phase II trial and received a total of 50 courses (median 2, range 1-6). Of these, dose reductions were performed in three courses because of leucopenia or stomatitis (1.5 mg m-2, one course; 1.45 mg m-2, two courses). Twenty-six patients were entered into the melanoma trial and received a total of 70 courses (median 2, range 1-12). No dose reductions were required. All patients were eligible for toxicity. Haematological toxicity included neutropenia CTC grade 3 (29/120 courses, 24.2%) and grade 4 (11/20 courses, 9.2%). Only drug-related CTC grade 1 thrombocytopenia was observed. Non-haematological toxicity included alopecia in all patients after two courses of treatment as well as CTC grade 3/4 stomatitis and asthenia. In the breast cancer study, one patient achieved a more than 50% tumour reduction after six cycles but was progressing after 6 weeks. Another patient showed a partial remission after the first course but was taken off the study because of CTC grade 3 skin toxicity. One patient was not evaluable for response (early death). No objective remissions were observed in 15 evaluable patients. In melanoma, no objective remissions were observed. We conclude that rhizoxin can be safely administered at 2.0 mg m-2 every 3 weeks. However, it has little activity in patients with advanced breast cancer and melanoma.


British Journal of Cancer (1996) 73, 397-399
© 1996 Stockton Press All rights reserved 0007-0920/96 $12.00

### Phase II clinical trials with rhizoxin in breast cancer and melanoma

A-R Hanauske[1], G Catimel[2], S Aamdal[3], W ten Bokkel Huinink[4], R Paridaens[5], N Pavlidis[6], SB Kaye[7], A te Velde[8], J Wanders[8], J Verweij[9] for the EORTC Early Clinical Trials Group

[1]*Division of Hematology and Oncology, I. Department of Medicine, Klinikum rechts der Isar der Technischen Universität München, Ismaninger Str. 22, D-81675 Munisch, Germany;* [2]*Centre Léon Bérard, Lyon, France;* [3]*The Norwegian Radium Hospital, Oslo, Norway;* [4]*The Netherlands Cancer Institute/Antoni van Leeuwenhoekhuis, Amsterdam, The Netherlands;* [5]*University Hospital, Clinic of Medical Oncology, Leuven, Belgium;* [6]*University of Ioaninna, Medical Oncology Section, Ioaninna, Greece,* [7]*University of Glasgow, Glasgow, UK;* [8]*EORTC NDDO, Amsterdam, The Netherlands;* [9]*Rotterdam Cancer Institute, Department of Medical Oncology, Rotterdam, The Netherlands.*

**Summary** Rhizoxin is a new anti-tumour agent isolated from the pathogenic fungus *Rhizopus chinensis*. It has shown broad activity against murine tumour models and is also active against vinca alkaloid-resistant cells. The purpose of our studies was to determine the clinical activity of this compound in patients with advanced breast cancer and melanoma. Based on the results of a phase I study, 2.0 mg $m^{-2}$ was administered as intravenous infusion over 5 min every 21 days. Nineteen patients were entered into the breast cancer phase II trial and received a total of 50 courses (median 2, range 1-6). Of these, dose reductions were performed in three courses because of leucopenia or stomatitis (1.5 mg $m^{-2}$, one course; 1.45 mg $m^{-2}$, two courses). Twenty-six patients were entered into the melanoma trial and received a total of 70 courses (median 2, range 1-12). No dose reductions were required. All patients were eligible for toxicity. Haematological toxicity included neutropenia CTC grade 3 (29/120 courses, 24.2%) and grade 4 (11/20 courses, 9.2%). Only drug-related CTC grade 1 thrombocytopenia was observed. Non-haematological toxicity included alopecia in all patients after two courses of treatment as well as CTC grade 3/4 stomatitis and asthenia. In the breast cancer study, one patient achieved a more than 50% tumour reduction after six cycles but was progressing after 6 weeks. Another patient showed a partial remission after the first course but was taken off the study because of CTC grade 3 skin toxicity. One patient was not evaluable for response (early death). No objective remissions were observed in 15 evaluable patients. In melanoma, no objective remissions were observed. We conclude that rhizoxin can be safely administered at 2.0 mg $m^{-2}$ every 3 weeks. However, it has little activity in patients with advanced breast cancer and melanoma.

**Keywords:** breast cancer; melanoma; rhizoxin; phase II trial

Rhizoxin (NSC 332.598; E 87/010) is a macrocyclic lactone antibiotic with antifungal and antineoplastic activity isolated from the pathogenic fungus *Rhizopus chinensis* (Iwasaki *et al.*, 1984, 1986; Tsuruo *et al.*, 1986). A recognised mechanism of action for rhizoxin includes binding to the tubulin $\beta$-chain at a site that is different from the vinblastine or colchicine binding sites but apparently identical with the binding site of maytansin (Takahashi *et al.*, 1987*a*, 1989; Bai *et al.*, 1990; Hamel, 1992). Subsequently, polymerisation of tubulin is effectively inhibited and depolymerisation of microtubuli promoted (Takahashi *et al.*, 1987*b*). By this mechanism, rhizoxin inhibits mitosis and lacks cross-resistance with vincristine or vinblastine (Otake, 1988). Rhizoxin has shown promising anti-tumour activity in a variety of experimental models *in vitro* and *in vivo*, including murine leukaemias, melanoma, sarcoma, breast, non-small and small-cell lung, colon and renal cancers (Kiyoto *et al.*, 1986; Hendriks *et al.*, 1992; Takigawa *et al.*, 1993). One clinical phase I trial has been completed and has shown a maximal tolerated dose of 2.6 mg $m^{-2}$ when the agent was administered as intravenous infusion over 5 min repeated every 3 weeks. Dose-limiting toxicities were mucositis, diarrhoea, and myelotoxicity. Other side-effects included malaise and phlebitis at the injection site. Minor tumour regressions were seen in two patients with advanced breast cancer in one phase I trial. The recommended dose with this schedule was 2.0 mg $m^{-2}$ (Bissett *et al.*, 1992; Graham *et al.*, 1992).

In view of the preclinical data indicating the potential for broad-spectrum activity, a co-ordinated phase II programme studying a number of tumor types was planned, involving the Cancer Research Campaign in the UK and the Early Clinical Trials Group (ECTG) of the EORTC. Within the ECTG, the studies were designed to determine whether partial or complete remissions could be achieved with rhizoxin in advanced breast cancer and melanoma and to determine their duration. In addition, assessment of the probability of an actual response rate and a more detailed characterisation of rhizoxin-related toxicities were intended.

### Patients and methods

### *Patients*

Eligibility criteria for both studies included: histologically or cytologically confirmed disease; at least one bidimensionally measurable lesion; age $\geqslant$18 years; adequate bone marrow function (leucocytes $\geqslant$4000 $\mu l^{-1}$, platelets $\geqslant$100 000 $\mu l^{-1}$); adequate renal function (serum creatinine $\leqslant$140 $\mu mol\ l^{-1}$ or creatinine clearance $\geqslant$60 ml $min^{-1}$; adequate liver function (serum bilirubin $\leqslant$26 $\mu mol\ l^{-1}$; SGPT and SGOT $\leqslant$ three times the upper limit of normal); WHO performance status $\leqslant$2; estimated life expectancy $\geqslant$3 months; no symptomatic brain or leptomeningeal disease; no other malignancies (exception: adequately treated cone-biopsied *in situ* carcinoma of the cervic uteri, basal or squamous cell carcinoma of the skin); no uncontrolled infection or other serious medical contidions; informed consent according to institutional guidelines. Pregnant or lactating females were not eligible for this study. Specific additional entry criteria for breast cancer patients were: not more than one prior chemotherapy regimen for advanced disease; discontinuation of chemotherapy or hormonal or radiation therapy at least 4 weeks before study entry (6 weeks in the case of prior mitomycin C or nitrosoureas. Specific additional entry criteria for melanoma patients were: no prior chemotherapy (exception adjuvant, local/extracorporal chemotherapy); prior immunotherapy with interleukin 2, interferon or other biological response modifiers was allowed.

Correspondence: A-R Hanauske
Received 1 June 1995; revised 15 August 1995; accepted 23 August 1995

*Treatment*

Rhizoxin was provided as lyophylisate by Fujisawa Pharmaceutical, Kashima, Japan. Immediately before use, the compound was reconstituted with a solution of 80% (v/v) propylene glycol and 20% (v/v) ethanol followed by water for injection to give a final concentration of 1 mg $ml^{-1}$ rhizoxin, 40% (v/v) propylene glycol, 10% (v/v) ethanol. Aliquots of 2 mg $m^{-2}$ of this solution were administered as intravenous infusion over 5 min repeated every 3 weeks. Doses of subsequent courses were adjusted according to toxicity. No dose escalation was planned. Treatment delay of more than 1 week, thrombocytopenic haemorrhage or febrile neutropenia requiring hospitilisation or non-haematologic toxicities greater than or equal to grade 3 resulted in a 25% dose reduction. Patients were treated until disease progression or unacceptable toxicity. For evaluation of response, patients had to receive at least two courses of therapy. Patients progressing after the first cycle were classified as early progression. Standard WHO criteria were applied for evaluation of response, NCI Common Toxicity Criteria were used for the grading of toxicities.

### Results

*Patients*

A total of 45 eligible patients were entered into two clinical phase II studies (melanoma: 26, breast: 19). Table I summarises the patients' characteristics. Two breast cancer patients entered were ineligible. One patient presented with liver metastases of less than 2.5 cm and the other patient had received two prior regimens for advanced disease. A total of 120 courses were administered. Breast cancer patients received a median of two courses with a range of 1–6. Melanoma patients received a median of two courses with a range of 1–12. Dose reductions were required in only three courses (all breast cancer patients). In these courses, doses were reduced by 25% owing to toxicity (haematologic and mucositis).

*Toxicities*

Myelosuppression was the predominant type of toxicity with CTC grade 3 or grade 4 occurring in approximately one-third of courses (Table II). One event of grade 4 thrombocytopenia was unrelated to treatment. This patient subsequently developed disease-related disseminated intravascular coagulation syndrome. Another event of grade 4 thrombocytopenia was considered treatment-related. Non-haematological toxicities are summarised in Table III. Alopecia was complete and almost universal after two courses of treatment. Nausea and vomiting were infrequent and mild.

*Response*

Of 19 patients entered into the breast cancer study, two were considered not evaluable for response and two not eligible. However, of these, one patient showed a partial reponse after the first treatment but had to be taken off the study owing to grade 3 skin toxicity, including a bullous exanthema involving the trunk, neck and arms. The patient received further treatment with the CMF regimen. A second patient was inevaluable for response due to early death. One patient had a partial response after six courses but was found to be progressive 6 weeks later. This patient was classified as having an overall response of 'no change'. Six additional patients were classified as 'no change' (median duration 12 weeks, range 12–16+ weeks) and eight patients had progressive disease. Therefore no formal objective remissions (95% CI: 0–22%) were seen in the 15 evaluable patients.

In the melanoma study, two patients were not evaluable for response, both were lost to follow-up after one and two cycles respectively. Four patients had 'no change' (median duration 12 weeks, range 6–36 weeks). Twenty patients were progressing while on treatment, including three patients with early progression. One patient was registered but as a result of rapid disease progression did not receive treatment with rhizoxin.

**Table I** Patients' characteristics

| *Parameter* | *No. of patients* Breast cancer | Melanoma |
|---|---|---|
| No. of eligable patients | 19 | 26 |
| Male/female | 0/17 | 16/10 |
| Age (years) median (range) | 56 (34–72) | 54 (24–76) |
| Performance status (WHO) | | |
| 0 | 7 | 15 |
| 1 | 7 | 9 |
| 2 | 3 | 2 |
| Prior therapy | | |
| Surgery | 17 | 26 |
| Chemotherapy | 16 | 2 |
| Immunotherapy | 3 | 0 |
| Hormonal therapy | 15 | 1 |
| Radiation | 15 | 4 |

**Table II** Treatment-related haematological toxicities per course (n = 120 cycles)

| *Parameter* | *NCI CTC grade* 1 | 2 | 3 | 4 | $\sum$ (%) |
|---|---|---|---|---|---|
| Leucopenia | 28 | 36 | 8 | 3 | 75 (63) |
| Neutropenia | 17 | 16 | 29 | 11 | 73 (61) |
| Anaemia | 40 | 7 | 1 | 0 | 48 (40) |
| Thrombyocytopenia | 5 | 0 | 0 | 1 | 6 (5) |

**Table III** Treatment-related haematological toxicities per course (n = 123 cycles)

| *Parameter* | *NCI CTC grade* 1 | 2 | 3 | 4 | $\sum$ (%) |
|---|---|---|---|---|---|
| Alopecia | 25 | 79 | – | – | 104 (85) |
| Asthenia | 30 | 11 | 2 | 1 | 44 (36) |
| Stomatitis | 13 | 23 | 2 | 1 | 39 (32) |
| Skin | 12 | 16 | 2 | 0 | 30 (24) |
| Nausea | 23 | 3 | 1 | 0 | 27 (20) |
| Allergy | 7 | 14 | 0 | 0 | 21 (17) |
| Diarrhoea | 15 | 4 | 0 | 0 | 19 (15) |
| Local | 10 | 7 | – | – | 17 (14) |
| Vomiting | 12 | 2 | 0 | 0 | 14 (11) |
| Fever | 6 | 6 | 0 | 0 | 12 (10) |
| Infection | 2 | 6 | 2 | 0 | 10 (8) |
| Liver | 7 | 2 | 0 | 0 | 9 (7) |

### Discussion

This report summarises two phase II studies with the new, tubulin-targeting agent rhizoxin that were conducted in patients with advanced breast cancer and melanoma. Although minor responses were observed in two breast cancer patients in a clinical phase I study with rhizoxin, our results indicate only marginal activity of this compound in advanced breast cancer (Bissett *et al.*, 1992). Of interest is, however, that both patients in the current studies had not received extensive prior treatment. One patient had achieved a partial response after four cycles of CMF 38 months before receiving rhizoxin. The other patient had only received hormonal and radiation therapy. All patients with 'no change' after rhizoxin had received prior anthrycycline-containing regimens. Also, 9 of 12 patients with progressive disease after rhizoxin had received prior anthracycline-containing regimens. These data indicate that chemonaive patients may be more sensitive to rhizoxin than pretreated patients and that further studies in minimally pretreated patients may be warranted. In the melanoma study no objective remissions or even short-lasting tumour regressions were noted. This indicates that rhizoxin is inactive against this tumour type, despite its activity in preclinical melanoma models. Similar results were obtained in phase II trials conducted by CRC in colon, renal and ovarian cancer (Kerr *et al.*, 1995). Further studies of this class of agents will depend on the identification of more effective analogues in preclinical models.

Haematological toxicities during these phase II studies were acceptable. The leading side-effect was neutropenia, which was accompanied by fever of unknown origin and infection in a total of four patients. In accordance with the observation of a previous phase I study, non-haematological toxicities mainly consisted of stomatitis and asthenia. However, a variety of other, less severe toxicities were causally related to treatment with rhizoxin, including alopecia, skin reactions, local phlebitis at the site of injection and gastrointestinal symptoms.

We conclude that the administration of rhizoxin at 2.0 mg $m^{-2}$ as intravenous bolus every 3 weeks is feasible and safe. However, clinical activity of this agent against advanced breast cancer is lacking. In addition, rhizoxin is clinically inactive against advanced melanoma.

### References

BAI RL, PETTIT GR AND HAMEL E. (1990). Binding of dolastatin 10 to tubulin at a distinct site for peptide antimitotic agents near the exchangeable nucleotide and vinca alkaloid sites. *J. Biol. Chem.*, **265**, 17141–17149.

BISSETT D, GRAHAM MA, SETANOIANS A, CHADWICK GA, WILSON P, KOIER I, HENRAR R, SCHWARTSMANN G, CASSIDY J, KAYE SB AND KERR DJ. (1992). Phase I and pharmacokinetic study of rhizoxin. *Cancer Res.*, **53**, 2894–2898.

GRAHAM MA, BISSETT D, SETANOIANS A, HAMILTON T, KERR DJ, HENRAR R AND KAYE SB. (1992). Preclinical and Phase I studies with rhizoxin to apply a pharmacokinetically guided dose-escalation scheme. *J. Natl Cancer Inst.*, **84**, 494–500.

HAMEL E. (1992). Natural products which interact with tubulin in the vinca domain: maytansine, rhizoxin, phomopsin A, dolastatins 10 and 15 and halichondrin B. *Pharmacol. Ther.*, **55**, 31–51.

HENDRIKS HR, PLOWMAN J, BERGER DP, PAULL KD, FIEBIG HH, FODSTAD O, DREEF VAN DER MEULEN HC, HENRAR RE, PINEDO HM AND SCHWARTSMANN G. (1992). Preclinical antitumour activity and animal toxicology studies of rhizoxin, a novel tubulin-interacting agent [see comments]. *Ann. Oncol.*, **3**, 755–763.

IWASAKI S, KOBAYASHI H, FURUKAWA J, NAMIKOSHI M, OKUDA S, SATO Z, MATSUDA I AND NODA T. (1984). Studies on macrocyclic lactone antibiotics. VII. Structure of a phytotoxin 'rhizoxin' produced by *Rhizopus chinensis*. *J. Antibiot. Tokyo.*, **37**, 354–362.

IWASAKI S, NAMIKOSHI M, KOBAYASHI H, FURUKAWA J, OKUDA S, ITAI A, KASUYA A, IITAKA Y AND SATO Z. (1986). Studies on macrocyclic lactone antibiotics. VIII. Absolute structures of rhizoxin and a related compound. *J. Antibiot. Tokyo.*, **39**, 424–429.

KERR DJ, RUSTIN GJ, KAYE SB, SELBY P, BLEEHEN NM, HARPER P AND BRAMPTON MH. (1995). Phase II trials of rhizoxin in advanced ovarian, colorectal and renal cancer. *Br. J. Cancer*, **72**, 1267–1269.

KIYOTO S, KAWAI Y, KAWAKITA T, KINO E, OKUHARA M, UCHIDA I, TANAKA H, HASHIMOTO M, TERANO H, KOHSAKA M, AOKI M AND IMANAKA H. (1986). A new anti-tumour complex, WF-1360, WF-1360A, B, C, D, E and F. *J. Antibiot. Tokyo.*, **39**, 762–772.

OTAKE N. (1988). [The structure and antitumour activity of anti-tumour antibiotics – recent progress]. *Gan. To. Kagaku. Ryoho.*, **15**, 369–379.

TAKAHASHI M, IWASAKI S, KOBAYASHI H, OKUDA S, MURAI T AND SATO Y. (1987*a*). Rhizoxin binding to tubulin at the maytansine-binding site. *Biochim. Biophys. Acta*, **926**, 215–223.

TAKAHASHI M, IWASAKI S, KOBAYASHI H, OKUDA S, MURAI T, SATO Y, HARAGUCHI HIRAOKA T AND NAGANO H. (1987*b*). Studies on macrocyclic lactone antibiotics. XI. Anti-mitotic and anti-tubulin activity of new anti-tumour antibiotics, rhizoxin and its homologues [published erratum appears in J Antibiot (Tokyo) 1987 Apr;40(4):following 565]. *J. Antibiot. Tokyo.*, **40**, 66–72.

TAKAHASHI M, KOBAYASHI H AND IWASAKI S. (1989). Rhizoxin resistant mutants with an altered beta-tubulin gene in *Aspergillus nidulans*. *Mol. Gen. Genet.*, **220**, 53–59.

TAKIGAWA N, OHNOSHI T, UEOKA H, HORIGUCHI T, KIURA K, TABATA M, SEGAWA Y, SHIBAYAMA T, GENBA K, MATSUMURA T. (1993). [Assessment of anti-tumour activity rhizoxin for human lung cancer cell lines: a potent new drug for drug-resistant lung cancer]. *Gan. To. Kagaku. Ryoho.*, **20**, 1221–1226.

TSURUO T, OH HARA T, IIDA H, TSUKAGOSHI S, SATO Z, MATSUDA I, IWASAKI S, OKUDA S, SHIMIZU F, SASAGAWA K, FUKAMI M, FUKUDA K AND ARAKAWA M. (1986). Rhizoxin, a macrocyclic lactone antibiotic, as a new anti-tumour agent against human and murine tumour cells and their vincristine-resistant sublines. *Cancer Res.*, **46**, 381–385.